# High genetic structure of *Spondias mombin* in Brazil revealed with SNP markers

**DOI:** 10.1590/1678-4685-GMB-2024-0030

**Published:** 2024-12-02

**Authors:** Allison Vieira da Silva, Caroline Bertocco Garcia, Igor Araújo Santos de Carvalho, Wellington Ferreira do Nascimento, Santiago Linorio Ferreyra Ramos, Doriane Picanço Rodrigues, Maria Imaculada Zucchi, Flaviane Malaquias Costa, Alessandro Alves-Pereira, Carlos Eduardo Batista, Edson Ferreira da Silva, Elizabeth Ann Veasey

**Affiliations:** 1 Universidade de São Paulo, Escola Superior de Agricultura “Luiz de Queiroz”, Departamento de Genética, Piracicaba, SP, Brazil.; 2 Universidade Federal do Maranhão, Centro de Ciências de Chapadinha, Chapadinha, MA, Brazil.; 3 Universidade Federal do Amazonas, Instituto Tecnológico de Ciências Exatas, Itacoatiara, AM, Brazil.; 4 Universidade Federal do Amazonas, Departamento de Genética, Manaus, AM, Brazil.; 5 Agência Paulista de Tecnologia dos Agronegócios, Piracicaba, SP, Brazil.; 6 Universidade Federal de Pernambuco, Departamento de Biologia, Recife, PE, Brazil.

**Keywords:** Amazon, conservation, genetic diversity, genomics, yellow mombin

## Abstract

*Spondias mombin* L. (Anacardiaceae) is an arboreal and allogamous fruit tree native from southern Mexico to southeastern Brazil, with great potential for economic exploitation. This study aimed to evaluate the structure and genomic diversity of yellow mombin accessions collected in nine locations in Brazil using Single Nucleotide Polymorphisms (SNP) markers. Significant genetic structure was observed in the discriminant analysis of principal components (DAPC) and dendrogram construction, in accordance with our hypotheses. The Mantel test identified a highly positive and significant correlation between geographic and genetic distances. The locations from the Amazon biome presented higher genetic diversity values when compared to those from the Atlantic Forest and Cerrado, which is expected considering the higher vulnerability of these biomes. However, although presenting greater genetic diversity, the Amazon biome showed positive inbreeding coefficients (*F*_*IS*_ ) in three of the four locations, ranging from 0.0855 to 0.2421, indicating a potential risk of genetic erosion, possibly related to the increased degradation of this biome in recent decades. The results obtained contribute to the understanding of the distribution of genetic variation and conservation status of yellow mombin in Brazil. They could also be used as a subsidy for developing conservation strategies and the genetic improvement of this species.

## Introduction

Several native fruit species with economic potential are found in Brazil. Among these, the yellow mombin (*Spondias mombin* L., Anacardiaceae) stands out for the agro-industrial value of its fruits, which can be consumed *in natura* or in the form of processed products (Silva Junior et al., 2004). Yellow mombin is a semi-domesticated species, native to areas ranging from southern Mexico to Paraguay and eastern Brazil, widely cultivated in the moist tropics (Mitchell and Daly, 2015). Its centers of diversity are the Atlantic Forest and the Western Amazon, more specifically the border regions between the State of Acre, in Brazil, and Peru and Bolivia (Fonseca *et al.,* 2017). The cultivation of this fruit tree was carried out by indigenous people in the Amazon even before the arrival of Europeans in the Americas in 1492 (Clement, 1999), and since then, it has been observed quite frequently in the North, Northeast, and Midwest regions of Brazil. In the Northeast region, it was described for the first time in 1587 in the Treaty Descriptive of Brazil by the Portuguese author Gabriel Soares de Souza (Sacramento and Souza, 2009).

It is an arboreal and erect species, reaching more than 20 m in height, with a trunk covered by very thick, grayish, rough, protruding bark and with cracks (Lorenzi, 1992). The leaves are compound, alternate, and imparipinnate, with 5 to 11 pairs of leaflets (Sacramento and Souza, 2009). *Spondias mombin* is a diploid with 2n = 16 = 32 chromosomes (Guerra, 1986; Almeida et al., 2007). It reproduces by cross-pollination performed mainly by bees, and also presents vegetative propagation (Ramos, 2009; Véras et al., 2017). The species is classified as andromonoecious (Ramos, 2009), having staminate and hermaphrodite flowers arranged in inflorescences of the terminal pyramidal panicles type (Coradin et al., 2022). Furthermore, it presents protandrous dichogamy, a high value of the pollen/ovule ratio (Ramos, 2009; Zortéa et al., 2019) and a tendency to self-incompatibility (Carneiro and Martins, 2012).

The yellow mombin fruits are bright yellow drupes when ripe, with juicy mesocarp (Lorenzi, 1992). The pulp has high levels of potassium, magnesium, phosphorus, and copper compared to other fruits consumed in Brazil; it has phenolic compounds, antioxidants, and carotenoids in greater concentration, which gives the fruit high nutritional value, and helps to prevent several diseases, including cardiovascular disease (Tiburski et al., 2011). In addition to the fruits, the leaves, seeds, stem bark, and even the flowers, have medicinal properties that have been used in ethnomedicine in cases of abortion, constipation, fever, gonorrhea, postpartum hemorrhage, digestive pain, diarrhea, dysentery, and wounds (Ogunro et al., 2023). Despite its importance, the yellow mombin is not yet cultivated on a significant commercial scale in Brazil (Sampaio et al., 2005), therefore there are no official records of the production and export of fresh fruits and its derivatives (Embrapa, 2022). However, it is estimated that its production is between 15 and 20 thousand tons of fruit per year, limited to the Northeast and North regions, with Bahia state responsible for 50% of the production. Under favorable growing conditions, each plant can produce around 40 kg, totaling approximately 6.2 tons of fruit per hectare (Embrapa, 2022). Thus, the characterization of diversity and genetic structure of natural populations of yellow mombin is fundamental to outlining conservation strategies and exploring the genetic resources of this species (Silva et al., 2017 a ). It is important to highlight that *S. mombin* is not considered threatened with extinction according to the criteria and categories of the International Union for Conservation of Nature and Natural Resources (IUCN) Red List (Zortéa, 2018). Although there is no current risk of extinction, the genetic diversity of the species may be compromised due to the destruction of its habitats (Mitchell and Daly, 2015), as its populations are found in fragmented areas with strong anthropogenic action (Zortéa *et al*., 2021).

For this purpose, previous studies used several markers, from morphological and physical/chemical data (Silva et al., 2014; Silva *et al.*, 2017a), as well as isoenzymatic (Silva *et al.*, 2009) and molecular markers, such as RAPD (Random Amplified Polymorphic DNA) (Lima et al., 2011), AFLP (Amplified Fragment Length Polymorphism) (Santos and Oliveira, 2008; Magalhães et al., 2013), ISSR (Inter Simple Sequence Repeat) (Silva *et al.,* 2017b), SSR (Simple Sequence Repeat) (Aguilar-Barajas et al., 2014; Cristóbal-Pérez et al., 2019; Zortéa et al., 2021), nrDNA (nuclear ribosomal DNA) and SNPs (Single Nuclear Polymorphism) (Nobre et al., 2018). However, none of these studies used such a geographically extensive sample, covering three biomes, as carried out in the present study.

SNP markers are the most abundant in the genome (Cortinovis et al., 2020). Also, in addition to the evaluation of neutral variation, they allow the study and identification of regions of the genome that are undergoing natural selection in the population, that is, regions directly associated with adaptation (Cortinovis *et al.*, 2020)⁠, presenting great potential in population genomics studies. SNP markers have been used in studies with fruit tree species from the Amazon, such as the cacao (*Theobroma cacao* L.) to understand the species’ domestication process (Cornejo et al., 2018), rubber tree [*Hevea brasiliensis*(Willd. ex Adr. de Juss.) Muell-Arg.] to analyze its population structure (Souza et al., 2018), as well as the juçara palm (*Euterpe edulis* Mart.) to analyze its genomic diversity (Alves-Pereira et al., 2019). SNP markers were also used to assess population structure and genetic diversity of native species such as *Acrocomia* spp. (Díaz et al., 2021; Oliveira et al., 2022), *Croton tetradenius* Baill. (Brito et al., 2021) and *Parkia platycephala* Benth. (Morais et al., 2023). In the genus *Spondias*, SNP markers have been used to test the hypothesis of a hybrid origin of the species *S. bahiensis* P. Carvalho, Van den Berg and M. Machado, as well as the species *S. mombin* and *S. tuberosa* Arruda (Nobre et al., 2018). SNP markers have also been used to evaluate the structure and genomic diversity of *S. tuberosa* from seven locations in the Caatinga biome (Nascimento et al., 2024).

The present study aims to characterize the diversity and genetic structure of *S. mombin* with a wide sampling, including nine locations originated from three biomes (Amazon, Cerrado, and Atlantic Forest) of Brazil, from SNP markers obtained with the genotyping-by-sequencing (GBS) technique. We expect to find in this study high genetic structure between locations and the DAPC (Discriminant Analysis of Principal Components) groups, and low to moderate levels of genetic diversity within locations due to genetic vulnerability of yellow mombin populations, threatened by anthropogenic activities and climate change. The results of this study will make it possible to identify locations with greater genetic vulnerability, provide information that could subsidize the management and conservation of genetic variability and contribute to developing future genetic improvement programs for the yellow mombin.

## Material and Methods

### Sampling, DNA extraction and quantification

**Figure 1 -:**
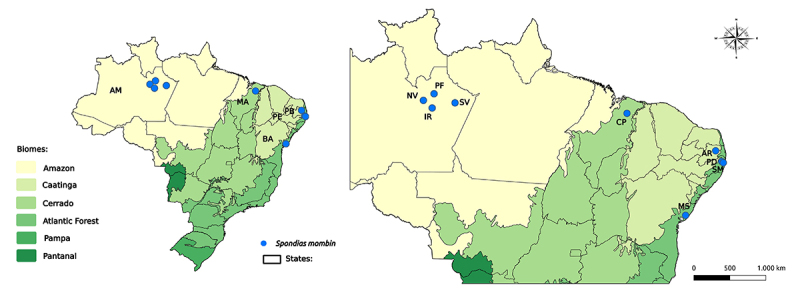
Map of Brazil with the sampling locations of *Spondias mombin* accessions in the States of Amazonas (AM), Maranhão (MA), Pernambuco (PE), Paraíba (PB), and Bahia (BA). The sampling location codes are described in Table 1.

Young leaves of yellow mombin adult individuals were collected from nine locations of three biomes: four from the Amazon biome, in the State of Amazonas-AM; four from the Atlantic Forest biome in Northeast Brazil, including two in the State of Pernambuco-PE, one in Paraíba-PB, and one in Bahia-BA; and one location from the Cerrado Biome, in the State of Maranhão-MA (Table 1; Figure 1). The collections in the Amazon biome were carried out along roadsides far from the urban centers of cities. It was possible to visualize that, although uninhabited, the sampling areas showed clear signs of anthropic action, with little presence of large trees. As for the collections in the Atlantic Forest and Cerrado biomes, they were made in homegardens, and the one in Bahia was carried out in a field area, near a rural community. This research was registered in the National System for the Management of Genetic Heritage and Associated Traditional Knowledge (SISGEN) (registration nº A3AF200). 

**Table 1 -:** Yellow mombin (*Spondias mombin*) locations used in the study including their origin (municipality/State), number of individuals sampled, biome, and geographic coordinates.

Location	Municipallity-State^a^	N	Collection Site	Biome	Latitude	Longitude
NV	Novo Airão-AM	5	Along roadsides	Amazon	2°37’20,8’’S	60°57’01,7’’W
IR	Iranduba-AM	2	Along roadsides	Amazon	3°17’03,4’’S	60°11’16,2’’W
PF	Presidente Figueiredo-AM	5	Along roadsides	Amazon	2°03’15,5’’S	60°01’41,8’’W
SV	Silves-AM	3	Along roadsides	Amazon	4°14’41,0”S	42°17’40,0”W
CP	Chapadinha-MA	3	Homegarden	Cerrado	3°44’43,0”S	43°22’20,3”W
PD	Paudalho-PE	6	Homegarden	Atlantic Forest	7°53’48,0”S	35°10’47,0”W
SM	São Lourenço da Mata-PE	6	Homegarden	Atlantic Forest	8°00’10,0”S	35º01’04,0”W
AR	Areia-PB	6	Homegarden	Atlantic Forest	6°59’19,6”S	35°44’11,6”W
MS	Mata de São João-BA	5	Field area, near a rural community	Atlantic Forest	12°29’19,6”S	37°59’42,8”W
Total		41				

^a^ States = AM - Amazonas, PE - Pernambuco, PB - Paraíba, and BA - Bahia

DNA was extracted from leaves using the protocol described by Inglis et al. (2018) with some modifications, including three to four prewashes using sorbitol wash buffer (100 mM Tris-HCl pH 8.0, 0.35 M Sorbitol, 5 mM EDTA pH 8.0, 1% (w/v) Polyvinylpyrrolidone (average molecular weight 40,000; PVP-40). Sample quantification was estimated using GelRed dye under UV in 1% agarose gels, comparing specific and known concentrations of lambda phage DNA (5, 20, 40, 80, and 160 ng), and validated with a high sensitivity fluorometer (Invitrogen), the Qubit 4, which presents a high level of accuracy. After quantification, DNA samples were normalized to a concentration of 20 ng μL^-1^ for GBS library preparation.

### Assembly of the genomic library and identification of SNPs

After digestion tests, the two enzymes to be used in yellow mombin were selected, *Pst*I and *Mse*I. The DNA samples were digested and had the barcode adapters attached to the fragments generated in the digestion with the aid of the enzyme NEB T4 DNA ligase #M0202 in NEB Buffer4 buffer in the presence of ATP. After the ligation reaction, the DNA fragments of each sample were bound to a specific barcode, allowing the multiplexing of the samples. The multiplex was amplified by PCR (polymerase chain reaction). The sequencing of PCR products was performed in a flowcell of a HiSeq2500 Illumina platform at the EcoMol, Genomics Center of Luiz de Queiroz College of Agriculture, University of São Paulo.

SNP markers were obtained using the Stacks v. 1.42 software (Catchen et al., 2011). The samples were initially submitted to the process_radtags stage, where low-quality sequences were eliminated. In this first step, demultiplexing is also performed, where the sequence of each individual is properly separated according to the unique barcodes. After this, the retained sequences were analyzed by the ustacks component with the parameters -m 3 (which defines the number of reads needed to form a stack), -M 2 (which specifies the number of mismatches allowed among stacks to be grouped into a locus), and -N 2 (the maximum distance allowed between primary stacks and secondary reads to be inserted into stacks). The next step was to create a catalog with all the possible identified loci and the presence of these loci in each individual. This step was performed by cstacks with the parameter -n 2 (which determines the number of mismatches between the loci of different samples for the formation of the catalog). After the formation of the catalog, two components were used to remove the loci with less probability (rxstacks, --lnl_lim -10), and to cross-reference information between the loci obtained for each individual and the loci of the catalog (sstacks). The population component was used for the final filtering of SNP markers. Only one SNP per tag was retained, with depth >= 3X, MAF (minimum allele frequency) >= 0.01, SNP present in at least 60% of samples within each sampling location, SNP present in at least five of the sampling locations. The datasets generated and analyzed during the current study are available in the Mendeley repository [https://data.mendeley.com/datasets/kzmzrdw5fr/1].

### Statistical analysis

SNP markers were analyzed with R software (R Core Team, 2020) from specialized packages. Genetic structuring analyses were performed using DAPC (Discriminant Analysis of Principal Components) (Jombart et al., 2010). DAPC was performed using the *adegenet* package (Jombart, 2008) in the R software. The analysis aims to identify groups based on genetic structure, minimizing variation within groups, and maximizing variation between groups. The number of regional groups (locations) was not provided in the analysis, and the optimal number of groups, which explains the data, was calculated by the k-means method, where several values ​​of k (groups) and several groupings were tested by using the Bayesian Information Criterion (BIC). The generated results were provided in a curve of BIC values ​​as a function of k, where the ideal value of groups was taken as the lowest value of the curve, from which the curve returns to its crescent. Clustering analyses were carried out from phylogenetic trees constructed by the *neighbor-joining* method without rooting, generated based on the genetic distance of Nei (1972) with the ape package (Paradis and Schliep, 2019).

Estimates of the basic parameters of genetic diversity, such as total number of alleles (*A*), observed heterozygosity (*H*_*O*_ ), expected heterozygosity (*H*_*E*_ ), and inbreeding coefficient (*F*_*IS*_ ) were generated with the hierfstat package (Goudet and Jombart, 2020). To generate the 95% confidence interval for the *F*_*IS*_ values, we used the boot.ppfis function from the hierfstat package with a hundred bootstraps. The Molecular Variance Analysis (AMOVA) was conducted using the poppr package (Kamvar et al., 2014 *,* 2015). Both the basic estimates of genetic diversity and the AMOVA were carried out in two situations, considering the locations (collection sites), and the groups formed from the DAPC analysis. With the hierfstat package, a pairwise *F*_*ST*_ matrix was generated, and the graphical visualization of the matrix was generated with the corrplot package (Wei and Simko, 2017). The Mantel test was performed to assess the correlation between genetic divergence between locations based on the pairwise *F*_*ST*_ value and the pairwise geographic distance between the locations in the ade4 package (Chessel et al., 2004; Dray and Dufour, 2007; Dray *et al.,* 2007; Bougeard and Dray, 2018; Thioulouse et al., 2018). The geographic distance matrix, generated from geographic coordinates, was built with the geodist package (Padgham and Sumner, 2020).

## Results

### Genetic diversity and structure of *Spondias mombin* locations

**Figure 2 -:**
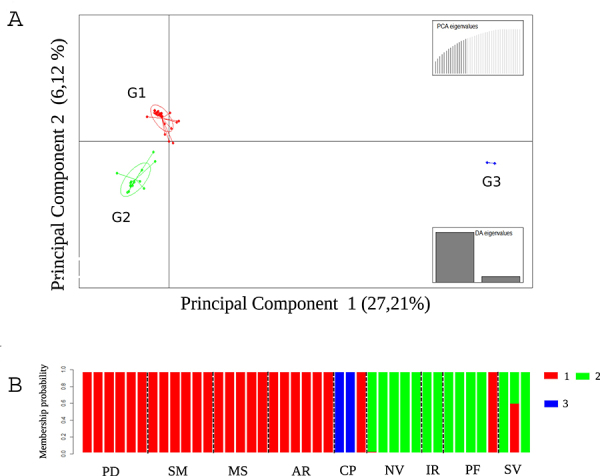
Discriminant Analysis of Principal Component (DAPC) performed with 41 yellow mombin (*Spondias mombin*) individuals and 2,003 SNP markers. A) A scatter plot based on two discriminant functions, with the three groups identified by the k-means method. B) Clustering probability analysis according to the results generated in the DAPC. Each delimited bar represents an individual, while the locations are represented by the interval formed between the dotted lines: PD (Paudalho - Pernambuco), SM (São Lourenço da Mata - Pernambuco), MS (Mata de São João - Bahia), AR (Areia - Paraíba) [Atlantic Forest biome]; CP (Chapadinha - Maranhão) [Cerrado biome]; and NV (Novo Airão -Amazonas), IR (Iranduba - Amazonas), PF (Presidente Figueiredo - Amazonas), and SV (Silves - Amazonas) [Amazon biome].

Structure and genetic diversity studies were carried out with 2,003 high quality SNP markers, used to evaluate 41 individuals of yellow mombin. The DAPC was performed using 15 principal components, which explained 77% of the total variation, and two discriminant functions. The optimal number of groups obtained from the *k-means* analysis corresponded to three groups (Figure 2 A ,B). Group G1, in red, was composed of 23 individuals from the locations of Paudalho-PE (PD), São Lourenço da Mata-PE (SM), Areia-PB (AR), Mata de São João-BA (MS), all from the Atlantic Forest biome; one accession from Chapadinha-MA (CP), belonging to the Cerrado biome; and one accession from Presidente Figueiredo-AM (PF), a location from the Amazon biome. Group G2, in green, was composed of 14 individuals from the State of Amazonas, at Iranduba (IR), Silves (SV), Presidente Figueiredo (PF), and Novo Airão (NV) locations, all from the Amazon biome. Group G3, in blue, comprises two of the three individuals from the Chapadinha-MA location in the Cerrado biome. The DAPC with k = 2 grouped the Chapadinha-MA location with the locations from the Atlantic Forest biome in the G1 group (Figure S1).

DAPC groups G1, predominantly from the Atlantic Forest biome, and G2, from the Amazon biome, were genetically closer than the G3 group, which was isolated from the other two groups. In the DAPC, with only one discriminant function, it was possible to visualize the grouping result in only one dimension (Figure S2). Groups G1 and G2 show an overlap area, with a greater distribution amplitude in group G2 (from the Amazon biome), demonstrating greater genetic variation within this group. The bar plot of the DAPC membership probabilities explicitly evidences the separation of individuals from the three groups, with only two exceptions: an accession from the Amazon biome (PF) and one from Chapadinha-MA (CP), within group G1, from the Atlantic Forest biome (Figure 2B). The G2 group contained only accessions collected in the State of Amazonas, from the Amazon biome. The G3 group was formed by two of the three individuals from Chapadinha, in the Cerrado biome. 

**Figure 3 -:**
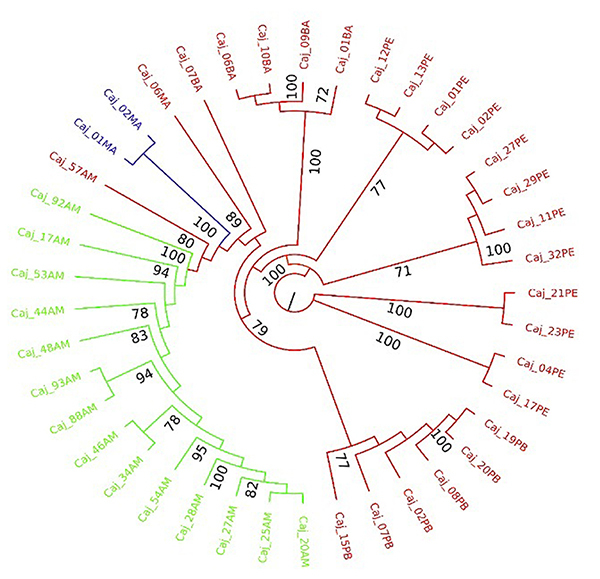
Cluster analysis using the *neighbor-joining* method and Nei’s distance (Nei, 1972) for the yellow mombin (*Spondias mombin*) accessions collected in the States of Amazonas (AM) (green, Amazon biome), Maranhão (MA) (blue, Cerrado biome), Bahia (BA), Pernambuco (PE) and Paraíba (PB) (red, Atlantic Forest biome). The colors green, blue and red are based on the DAPC groups.

Cluster analysis, performed using the *neighbor-joining* method and Nei’s distance (Nei, 1972), shows the position of each accession regarding the groups identified by the DAPC analysis (Figure 3), confirming the clustering probability analysis according to the results generated in the DAPC. In this analysis, the Amazon biome locations (green) were separated from the Atlantic Forest biome locations (red), with the Cerrado location of Chapadinha (blue) in between the other two biomes. Also, it can be noticed that the third accession of Chapadinha, classified in the G1 group (Caj_06MA), is genetically very close to the other two Chapadinha accessions of group G3 in the DAPC. Another clustering analysis was performed using the *neighbor-joining* method (Figure S3). This tree was built without defined rooting, not for evolutionary inferences but for understanding how the groups defined in the DAPC relate to each other. Five distinct groups were formed: groups I (location from Bahia, BA), II (locations from Pernambuco, PE), and III (location from Paraíba, PB), which are equivalent to group G1 in the DAPC, formed three well-defined groups from the Atlantic Forest biome. Group IV, which is equivalent to group G3 in the DAPC, was formed by two of the three accessions from the Maranhão (MA) location, Cerrado biome, and was placed between the Atlantic Forest biome groups (I, II and III) and group V, formed by the Amazon biome locations, from Amazonas (AM), equivalent to G2. 

In the pairwise analysis of genetic divergence (*F*_*ST*_ ), values greater than 0.25 were observed in all comparisons. The highest *F*_*ST*_ values were identified between groups G1 and G3 (0.37), and G2 and G3 (0.36), and the lowest *F*_*ST*_ value (0.33) was observed between groups G1 and G2 (Table 2). AMOVA provided similar results for the DAPC groups and locations, both significant at 5% probability (Table 3). In both scenarios, the highest percentage of variation occurred within DAPC groups and within locations, 59.3% and 61.4%, respectively.

**Table 2 -:** Result of pairwise *F*st analysis between groups identified by DAPC for *Spondias mombin* species. Group G1 is formed by the locations in the states of Pernambuco, Bahia, and Paraíba, Atlantic Forest biome. Group G2 is composed by the Amazon biome locations, from the State of Amazonas. Group G3 is formed by two accessions from the Cerrado biome location in the State of Maranhão.

F_*st*_	G1	G2	G3
G1	0.00		
G2	0.33	0.00	
G3	0.37	0.36	0.00

**Table 3 -:** Results generated by AMOVA to identify the sources of genetic variation among and within the groups identified in the DAPC and the locations of *Spondias mombin.*

Source of variation	Degree of freedom	Sums of square	Coefficient of variation	Percent variation	F statistics
Among groups	2	2,481.53	104.30	40.69	0.4070*
Within groups	38	5,775.89	151.99	59.31	
Among locations	8	12,636.86	259.25	38.56	0.3856*
Within locations	32	13,215.64	412.99	61.44	

*Significant at *p*-value < 0.05.

**Figure 4 -:**
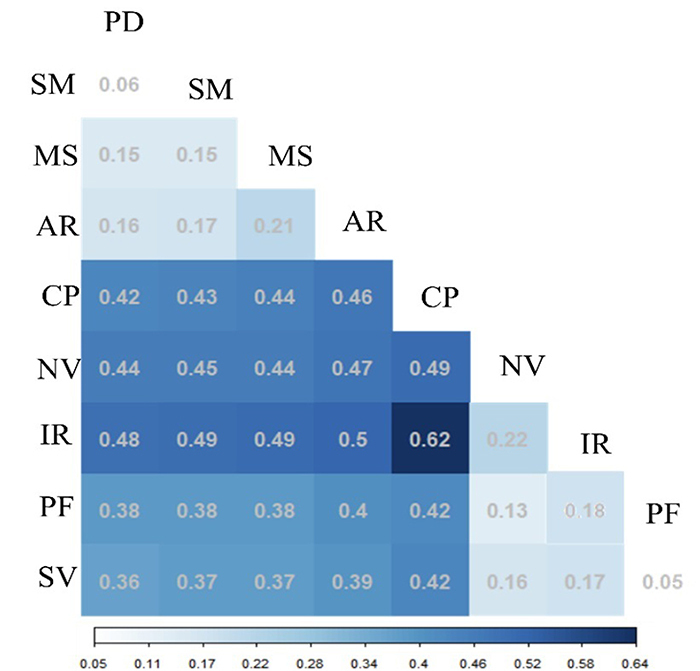
Results obtained from the pairwise analysis of *F*_*ST*_ between yellow mombin (*Spondias mombin*) locations: PD (Paudalho-Pernambuco), SM (São Lourenço da Mata-Pernambuco), MS (Mata de São João-Bahia), AR (Areia-Paraíba), CP (Chapadinha-Maranhão), NV (Novo Airão-Amazonas), IR (Iranduba-Amazonas), PF (Presidente Figueiredo-Amazonas), and SV (Silves-Amazonas). All values are significant at *p*-value < 0.05.

The pairwise *F*_*ST*_ matrix, generated with the nine locations (collection sites), enabled the comparison between locations (Figure 4; Table S1). When the locations from the Atlantic Forest biome were compared to each other, the two locations from the State of Pernambuco, São Lourenço da Mata (SM) and Paudalho (PD), presented the lowest *F*_*ST*_ value (0.06). The highest *F*_*ST*_ value within this biome was between Areia (AR), Paraíba, and Mata de São João (MS), Bahia, generating an *F*_*ST*_ of 0.21. Within the Amazon biome locations, the lowest *F*_*ST*_ value (0.05) was obtained between Silves (SV) and Presidente Figueiredo (PF). The greatest genetic divergence among the Amazonian locations was between Iranduba (IR) and Novo Airão (NV), with an *F*_*ST*_ value of 0.22, indicating a high genetic divergence between these locations. The geographic distance matrix constructed from the geographic coordinates presents distance values between the locations varying from 21.4 km to 2,934.4 km (Table S1). The Mantel test identified a positive and significant correlation (r = 0.78; p-value < 0.001) between the geographic distance among locations and the values of genetic divergence (*F*_*ST*_ ), showing the occurrence of isolation by distance for the yellow mombin.

Two scenarios were considered to obtain the basic genetic diversity parameters. The first considered the groups identified in the DAPC, and the second considered the locations (collection sites) (Table 4). The G2 group (Amazon biome) had the highest number of alleles (*A* = 3,547) as well as the highest observed (*Ho* = 0,1568) and expected heterozygosity (*He* = 0.2031), and it was 37% genetically more diverse than the G1 group and 70% more diverse than the G3 group, presenting 649 more alleles than the G1 group and 1307 more than the G3 group. The result presented by the G2 group agrees with the inference obtained in the DAPC, showing a wider distribution, followed by the G1 group. Group G3 had the lowest number of alleles (*A* = 2,240) and lower gene diversity (*He* = 0.0594). However, the G2 group had the highest value for the inbreeding coefficient (*F*_*IS*_ = 0.2280), almost double in relation to the G1 group. The G3 group had a negative value (*F*_*IS*_ = -0.8529). 

**Table 4 -:** Genetic diversity analysis carried out for the groups identified in the DAPC and the locations of yellow mombin (*Spondias mombin*): PD (Paudalho-Pernambuco), SM (São Lourenço da Mata-Pernambuco), MS (Mata de São João-Bahia), AR (Areia-Paraíba) [Atlantic Forest biome]; CP (Chapadinha-Maranhão) [Cerrado biome]; and NV (Novo Airão-Amazonas), IR (Iranduba-Amazonas), PF (Presidente Figueiredo-Amazonas), and SV (Silves-Amazonas) [Amazon biome].

Groups	N^*a*^	A	Ho	He	F_*IS*_	F_*IS*_ CI95%
G1	25	2,898	0.1128	0.1279	0.1187	0.0979: 0.1402
G2	14	3,547	0.1568	0.2031	0.2280	0.2103: 0.2540
G3	2	2,240	0.1101	0.0594	-0.8529	-0.8959: -0.8035
Average	-	2,895	0.1266	0.1301	-0.5062	
Locations (Atlantic Forest biome)						
PD	6	2,624	0.1205	0.1184	-0.0177	-0.0568: 0.0143
SM	6	2,624	0.1254	0.1178	-0.0649	-0.1057: -0.0210
AR	6	2,492	0.0833	0.0990	0.1581	0.1015: 0.2070
MS	5	2,562	0.1213	0.1170	-0.0371	-0.0825: 0.0012
Average	-	2,576	0.1126	0.1131	-0.0096	
(Cerrado biome)						
CP	3	2,244	0.1100	0.0606	-0.8146	-0.8585: -0.7567
(Amazon biome)						
IR	2	2,447	0.1790	0.1094	-0.6362	-0.7962: -0.4762
SV	3	2,778	0.1873	0.2092	0.1045	0.0356: 0.1195
NV	5	2,806	0.1509	0.1650	0.0855	0.0381: 0.1260
PF	5	2,856	0.1505	0.1986	0.2421	0.1885: 0.2700
Average	-	2,722	0.1669	0.1706	-0.0510	

^a^ Number of individuals (N), total number of alleles (*A*), observed heterozygosity (*Ho*), expected heterozygosity (*He*), and inbreeding coefficient (*F*_*IS*_ ).

When considering the locations, the result was similar to that obtained with the DAPC groups. The Amazon biome locations showed, on average, higher genetic diversity values when compared to the locations from the Atlantic Forest and Cerrado biomes (Table 4). The Iranduba population presented divergent values from the other locations in the Amazonas State, with lower number of alleles and expected heterozygosity, and a negative inbreeding coefficient. The locations from the Atlantic Forest biome showed similar values for the genetic diversity parameters, except for Areia, Paraíba, with lower heterozygosity values and a positive inbreeding coefficient. The Cerrado location, in Chapadinha, showed the lowest number of alleles and *He* among all the evaluated populations. 

## Discussion

The results clearly showed the genetic structure among the locations sampled in this study and the DAPC groups, in accordance with our initial hypothesis, and a trend of grouping yellow mombin locations by State. The pairwise *F*_*ST*_ matrix constructed with the groups identified in the DAPC analysis showed *F*_*ST*_ > 0.25 in all combinations between the three groups. *F*_*ST*_ values of this magnitude indicate very high genetic differentiation based on the scale proposed by Hartl and Clark (2007). The original *F*_*ST*_ values utilized by Hartl and Clark (2007) were derived from previous studies using other traditional markers, which can exhibit different levels of polymorphism and mutation rates compared to SNPs. Consequently, direct comparisons should be made with caution, and interpretations should consider the specific characteristics and limitations of the SNP data. These references are still used for studies of the genetic structure of native species populations using SNPs markers, such as those developed by Garcia et al. (2024) who observed a clear separation between locations from the Amazon, Atlantic Forest and Cerrado biomes when studying populations of *Platonia insignis* Mart.; and Costa et al. (2022) who suggested low to high genetic differentiation between populations of *Copernicia prunifera* (Miller) H. E. Moore from the Caatinga. Both studies used SNP markers and obtained high *F*_*ST*_ (ranging from 0.59 to 0.68) and moderate to high (ranging from 0.118 to 0.20), respectively.

Also, the pairwise *F*_*ST*_ matrix among the nine locations studied allowed the identification of the lower divergence between locations from the same biome. The *F*_*ST*_ values between locations of the same biome indicate genetic divergence of a moderate (between 0.05 and 0.15) to a high degree (between 0.15 and 0.25) (Hartl and Clark, 2007). However, in all scenarios in which locations from two biomes are compared, the values ​​obtained indicate very high genetic divergence, with *F*_*ST*_ ranging from 0.36 to 0.50. Garcia et al. (2024) and Nascimento et al. (2021) also observed a clear separation between locations from the Amazon biome (North region) and Atlantic Forest and Cerrado biomes (Northeast region) when studying populations of bacuri tree (*Platonia insignis* Mart.) with SNP and SSR markers, respectively.

AMOVA showed that the greatest source of genetic variation is found among accessions within locations (61.4%) and within DAPC groups (59.3%) than among locations (38.6%) and among DAPC groups (40.7%). Higher intrapopulation than interpopulation genetic variation is expected in allogamous perennial species with self-incompatibility since most variation occurs between individuals within populations (Hamrick et al., 1992; Paschoa et al., 2018; Francisconi et al., 2021). Yellow mombin is an allogamous species and depends on cross-pollination for greater reproductive success, showing protandrous dichogamy and a high value for the pollen/ovule ratio (Ramos, 2009). 

Yellow mombin fruits are appreciated as juice and drinks and represent an important source of income in many regions. A study by Silva et al. (2009) with populations of this species in Northeast Brazil suggested that commercialization and the exchange of fruits among communities are important mechanisms acting in the gene flow between distant populations of this species. The dispersion of fruits over long distances are factors that favor a greater concentration of genetic variation within populations of tree species (Hamrick et al., 1992). Although intra-location or intra-group variation was the major source of variation detected in the AMOVA, the percentage of interpopulation variation was also quite expressive. F statistics indicate significant genetic divergence between the DAPC groups and the studied locations, results similar to those identified by Magalhães et al. (2013), who analyzed yellow mombin populations in the Amazon using AFLP markers and identified 47.2% of the variation between populations, while 52.8% was found within the studied populations. Two studies evaluating populations of *S. mombin* originating from the State of Mato Grosso, in the Midwest region of Brazil, also observed that most of the genetic variation was found within populations: 77.4% by Silva *et al.* (2017a) with ISSR markers and 90.6% by Zortéa et al. (2021) with SSR markers. In studies carried out with other tropical fruit species, Surapaneni et al. (2013) also observed, with SSR markers, that 62.2% of the genetic variation in *Mangifera indica* L. originated from variation among individuals within populations, while Santos et al. (2019) observed, using ISSR markers, that 78% of the variation in *Anacardium occidentale* L. originated within populations. Therefore, the execution of conservation actions or the use of the genetic diversity of yellow mombin must consider greater diversity within populations or within different biomes than between them.

Considering the genetic diversity parameters, the Amazon biome locations from the State of Amazonas showed the highest values, which can be expected as this region encompasses the center of diversity for the species (Fonseca *et al.*, 2017). However, from the results obtained with all nine locations, a proper classification of the genetic diversity values for *S. mombin* would be low to moderate values, which is in accordance with our hypothesis. Comparing the genetic diversity parameters with the literature, up to our knowledge, no other study has used SNP markers to assess genetic structure and diversity of yellow mombin populations. However, a study carried out with SNP markers by our group found higher genetic diversity values for *S. tuberosa* accessions (*H*_*O*_ = 0.221 and *H*_*E*_ = 0.199, on average), from the Caatinga biome (Nascimento et al., 2024). Also based on SNP markers, higher genetic diversity values were found for cacao (*Theobroma cacao* L.) varieties in Honduras and Nicaragua (*H*_*O*_ = 0.206; *H*_*E*_ = 0.367, on average) (Ji et al., 2013), as well as for populations of *Parkia platycephala* Benth. located at the Sete Cidades National Park, in the state of Piauí (*H*_*O*_ = 0.29; *H*_*E*_ = 0.29, on average), in a transition zone between Caatinga and Cerrado (Morais et al., 2023).

Another reason for the lower genetic diversity found in the four Atlantic Forest locations, is that this biome is one of the most degraded in Brazil, with 28% of the natural vegetation remaining (Rezende et al., 2018; Guerra et al., 2020). The major causes of degradation in both the Atlantic Forest and Cerrado biomes are agriculture and livestock (Guerra *et al*., 2020). Two yellow mombin locations (Chapadinha and Areia, from the Cerrado and Atlantic Forest biomes, respectively) showed low genetic diversity values that may be related to the reduction in the number of individuals in these sample sites, which appear to suffer more intense anthropic action. Although Chapadinha, from the State of Maranhão, occurs in a border area of Cerrado and does not represent the entire biome, this location showed low genetic diversity levels when compared to the Amazon locations, and observed heterozygosity (*Ho*) higher than the expected heterozygosity (*He*), which indicates the recent introduction of alleles in the population. Also, although represented by only three accessions, this location is clearly atypical, markedly different from the rest of individuals and locations analyzed. This location belonged, in the past, to an environmental protection area (PA). It occurs in the extractive reserve of Chapada Limpa, which is subjected to urban advance. Even though it belonged to a PA, the use and occupation of these areas does not prevent irregular activities from taking place (Morelli et al., 2009), such as logging and the opening of swiddens for agriculture, which may lead to significant losses of genetic diversity. The Areia location is a highland swamp located in the State of Paraíba, typical of the Atlantic Forest biome, although this area is surrounded by the Caatinga biome (Marques et al., 2014), that also showed a genetic diversity value below the expected average and, in addition, the highest inbreeding coefficient (*F*_*IS*_ = 0.1581), the only positive one among the Atlantic Forest locations. The high-altitude microclimate of the region (highland swamps) could interfere with the local flora. However, this location has great potential for supplying genes related to water stress. Thus, the northeastern semi-arid region is expected to significantly influence its flora. Therefore, we consider it extremely important to maintain diversity in this location, increasing the chances of obtaining the greatest possible number of genes adapted to such adverse edaphoclimatic conditions.

It would be interesting at this point to consider the possible differences among the anthropic action in each collection site. The Amazon biome accessions were collected along roadsides in all the locations, while the Atlantic Forest and Cerrado biome accessions, with one exception, were collected in homegardens, where a higher anthropic action might be expected. The exception was at Mata de São João location, in Bahia, where yellow mombin individuals were collected at a field area, but near a rural community. No differences were found for this location as far as genetic diversity when compared to the other Atlantic Forest biome locations. Considering the inbreeding coefficient, except for the Iranduba location, all other Amazon biome locations showed positive values for the inbreeding coefficient. This may be related to the degree of preservation of this biome, which despite showing a 22% reduction in the deforestation rate between 2022 and 2023 (INPE, 2023), is still considered insufficient due to the preservation policies of the Amazon Forest having been very weakened in the last years. The main drivers of Amazonian habitat destruction and degradation are land-use changes (land clearing, wildfires, and soil erosion), water-use changes (damming and fragmenting rivers and increased sedimentation from deforestation), and aridification from global climate change. Additional important threats come from overhunting and overfishing, introduction of invasive exotic species, and pollution from the mining of minerals and hydrocarbons (Albert et al., 2023). Thus, the state of alert is increased for the yellow mombin in the region, which, despite having greater genetic diversity, showed a higher inbreeding coefficient and risk of genetic erosion.

A high number of *S. mombin* accessions are maintained in germplasm banks in Brazil, all maintained *ex situ* (Ramos, 2009; Portal Alelo Recursos Genéticos, 2022). However, only one accession from the State of Amazonas was verified in the existing germplasm banks. There is an urgent need to include accessions from the northern Amazon biome region in the existing germplasm bank or create new banks for the conservation of the genetic diversity of that region, maximizing the chances of preserving the greatest possible genetic diversity of the species, highlighting that in the present study, the locations from the Amazon biome presented the highest genetic diversity. The creation of an *in situ* genetic reserve is also indicated. *In situ* preservation favors the maintenance of fauna and flora in the area to be preserved, allowing the species to co-evolve in the habitat under the same selection pressures to which its pollinators and pests are subjected (Ramos *et al.,* 2015). Considering the wide area of occurrence of the yellow mombins, the creation of protected areas and the expansion of existing ones where this species occurs may be the most economical alternative. According to the results obtained in this study, the species presents a genetic structure regarding geographic distance, with a high correlation between geographic distance and genetic differentiation. 

In conclusion, we demonstrated that *Spondias mombin* presents a pattern of genetic structure of isolation by distance, with limited gene flow between locations from different biomes. Greater genetic diversity was found in the locations from the Amazon biome region. On the other hand, the Amazon biome locations showed higher values for the inbreeding coefficient when compared to the locations from the other two biomes. From a conservation point of view, the species has the potential for *in situ* conservation with populations that show reasonable levels of genetic diversity. Curators of *ex situ* germplasm banks for the species should consider inserting accessions from different regions of the country in the collection, mainly from the Amazon biome, which presented higher levels of genetic diversity, increasing efficiency in the conservation of the genetic diversity of the species. The study was carried out using a broad sampling of the geographic distribution of the species in the country, and the wide coverage of genomic diversity contributed significantly to the understanding of the current state of conservation of the species and will enable breeders to devise strategies for its conservation and genetic improvement.

## Supplementary material

The following online material is available for this article:

Table S1 - On the upper diagonal, the pairwise matrix of geographical distance was calculated between the locations, and on the lower diagonal, the *F*_*ST*_ values were calculated between the locations.

Figure S1 - DAPC analysis generated with K = 2 and 15 principal components.

Figure S2 - Discriminant Analysis of Principal Component (DAPC) from the first discriminant function to better understand the density and overlap between groups.

Figure S3 - Cluster analysis from the phylogenetic tree built by the *neighbor-joining* method based on the groups identified by the DAPC analysis for the yellow mombin (*Spondias mombin*) locations from the States of Amazonas (AM), Maranhão (MA), Bahia (BA), Pernambuco (PE) and Paraíba (PB).
