# Supplementary material for: High genetic structure of Spondias mombin in Brazil revealed with SNP markers
Source: Genet Mol Biol. 2024 Dec 2;47(4):e20240030. doi: 10.1590/1678-4685-GMB-2024-0030 (PMC11719815; doi:10.1590/1678-4685-GMB-2024-0030)
Supplement: Table S1 - [file 1415-4757-GMB-47-4-e20240030-s1.pdf]

Genetics and Molecular Biology
https://doi.org/10.1590/1678-4685-GMB-2024-0030

Supplementary Material to "High genetic structure of *Spondias mombin* in Brazil revealed with SNP markers"

Table S1 - On the upper diagonal, the pairwise matrix of geographical distance was calculated between the locations, and on the lower diagonal, the *F*st values were calculated between the locations. The distance values shown are in kilometers. The locations are represented by the abbreviations AR (Areia-Paraíba), CP (Chapadinha-Maranhão), IR (Iranduba-Amazonas), MS (Mata de São João-Bahia), NV (Novo Airão-Amazonas), PD (Paudalho-Pernambuco), PF (Presidente Figueiredo-Amazonas), SM (São Lourenço da Mata-Pernambuco), and SV (Silves-Amazonas).

Populations	AR	CP	IR	MS	NV	PD	PF	SM	SV
AR		919.04	2,741.17	656.84	2,837.75	117.73	2,749.53	137.37	2,553.47
CP	0.46		1,869.07	1,133.84	1,957.92	1,016.66	1,861.16	1,037.87	1,672.22
IR	0.51	0.62		2,647.72	112.19	2,816.34	137.16	2,835.82	205.18
MS	0.21	0.44	0.50		2,755.27	594.25	2,689.56	593.78	2,487.38
NV	0.47	0.49	0.23	0.44		2,914.74	120.44	2,934.39	285.75
PD	0.16	0.43	0.49	0.15	0.45		2,829.20	21.36	2,631.60
PF	0.41	0.44	0.19	0.39	0.16	0.38		2,849.09	202.19
SM	0.17	0.43	0.49	0.15	0.45	0.06	0.39		2,651.36
SV	0.39	0.43	0.21	0.37	0.17	0.36	0.08	0.37	
