# Supplementary material for: High genetic structure of Spondias mombin in Brazil revealed with SNP markers
Source: Genet Mol Biol. 2024 Dec 2;47(4):e20240030. doi: 10.1590/1678-4685-GMB-2024-0030 (PMC11719815; doi:10.1590/1678-4685-GMB-2024-0030)
Supplement: Figure S1 - [file 1415-4757-GMB-47-4-e20240030-s2.pdf]

Genetics and Molecular Biology
https://doi.org/10.1590/1678-4685-GMB-2024-0030

Supplementary Material to "High genetic structure of *Spondias mombin* in Brazil revealed with SNP markers"

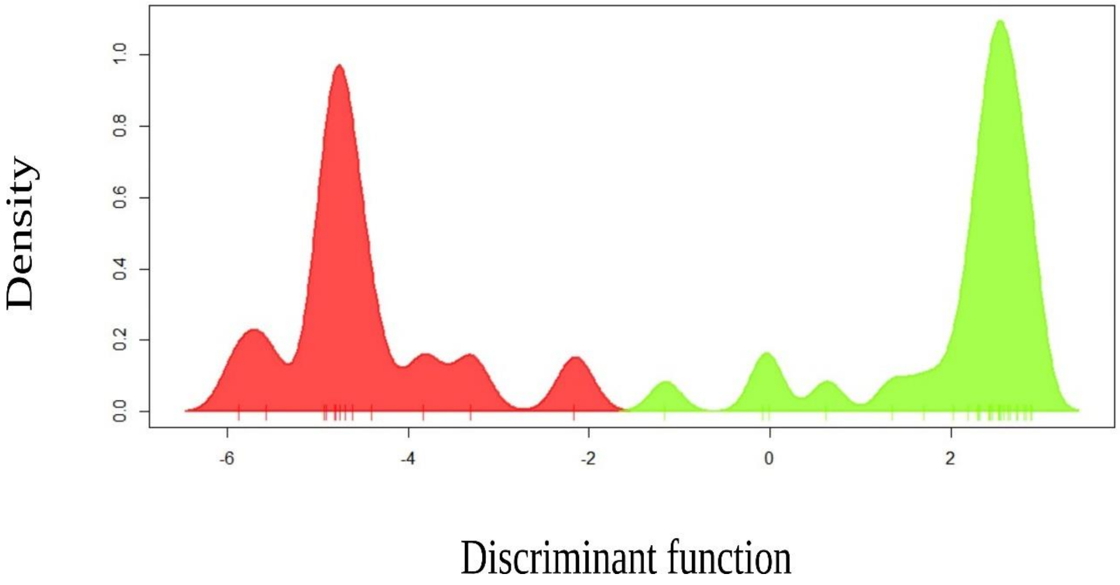

Figure S1 - DAPC analysis generated with K = 2 and 15 principal components. The red group corresponds to the G1 group formed by the yellow mombin (*Spondias mombin*) locations from Paudalho and São Lourenço da Mata - Pernambuco, Mata de São João - Bahia, Areia - Paraíba and Chapadinha - Maranhão. The green group corresponds to the G2 group composed of Iranduba, Novo Airão, Presidente Figueiredo, and Silves populations, all from the State of Amazonas in the Amazon biome.
