# Supplementary material for: High genetic structure of Spondias mombin in Brazil revealed with SNP markers
Source: Genet Mol Biol. 2024 Dec 2;47(4):e20240030. doi: 10.1590/1678-4685-GMB-2024-0030 (PMC11719815; doi:10.1590/1678-4685-GMB-2024-0030)
Supplement: Figure S2 - [file 1415-4757-GMB-47-4-e20240030-s3.pdf]

Genetics and Molecular Biology
https://doi.org/10.1590/1678-4685-GMB-2024-0030

Supplementary Material to "High genetic structure of *Spondias mombin* in Brazil revealed with SNP markers"

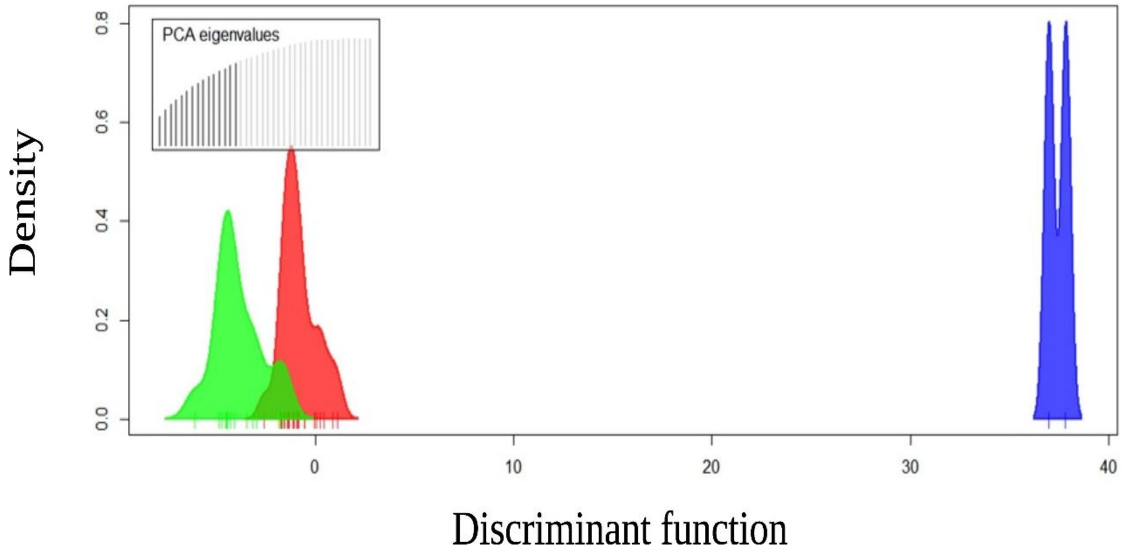

Figure S2 - Discriminant Analysis of Principal Component (DAPC) from the first discriminant function to better understand the density and overlap between groups. The G1 group is represented in red, the G2 group is represented in green, and the G3 group is represented in blue. This analysis was performed with 41 yellow mombin (*Spondias mombin*) individuals and 2,003 SNP markers.
