# Supplementary material for: High genetic structure of Spondias mombin in Brazil revealed with SNP markers
Source: Genet Mol Biol. 2024 Dec 2;47(4):e20240030. doi: 10.1590/1678-4685-GMB-2024-0030 (PMC11719815; doi:10.1590/1678-4685-GMB-2024-0030)
Supplement: Figure S3 - [file 1415-4757-GMB-47-4-e20240030-s4.pdf]

Genetics and Molecular Biology
https://doi.org/10.1590/1678-4685-GMB-2024-0030

Supplementary Material to "High genetic structure of *Spondias mombin* in Brazil revealed with SNP markers"

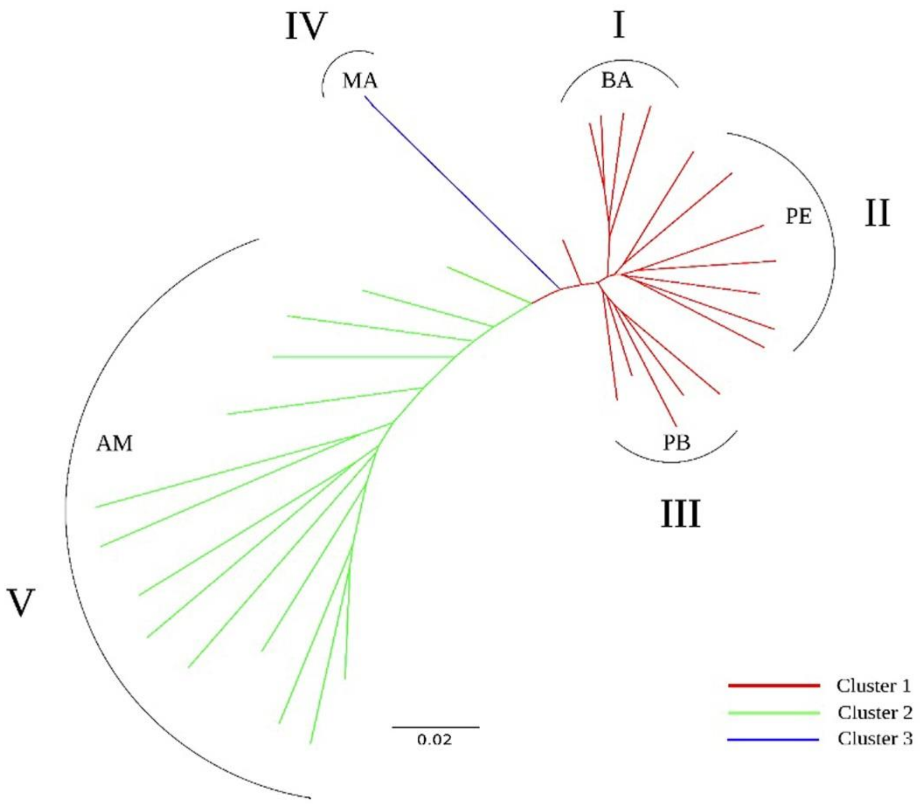

Figure S3 - Cluster analysis from the phylogenetic tree built by the *neighbor-joining* method based on the groups identified by the DAPC analysis for the yellow mombin (*Spondias mombin*) locations from the States of Amazonas (AM), Maranhão (MA), Bahia (BA), Pernambuco (PE) and Paraíba (PB). The Roman numerals (I, II, III, IV, and V) represent the groups obtained in this analysis, while the colors represent the groups identified by the DAPC.
